# Genetic and epigenetic instability as an underlying driver of progression and aggressive behavior in IDH-mutant astrocytoma

**DOI:** 10.1007/s00401-024-02761-7

**Published:** 2024-07-16

**Authors:** Timothy E. Richardson, Jamie M. Walker, Dolores Hambardzumyan, Steven Brem, Kimmo J. Hatanpaa, Mariano S. Viapiano, Balagopal Pai, Melissa Umphlett, Oren J. Becher, Matija Snuderl, Samuel K. McBrayer, Kalil G. Abdullah, Nadejda M. Tsankova

**Affiliations:** 1 Department of Pathology, Molecular and Cell-Based Medicine, Icahn School of Medicine at Mount Sinai, 1468 Madison Avenue, Annenberg Building, 15.238, New York, NY 10029 USA; 2 Nash Family Department of Neuroscience, Icahn School of Medicine at Mount Sinai, New York, NY 10029 USA; 3 Department of Oncological Sciences, The Tisch Cancer Institute, Mount Sinai Icahn School of Medicine, New York, NY 10029 USA; 4 Department of Neurosurgery, Mount Sinai Icahn School of Medicine, New York, NY 10029 USA; 5 Department of Neurosurgery, Perelman School of Medicine, University of Pennsylvania, Philadelphia, PA 19104 USA; 6 Glioblastoma Translational Center of Excellence, Abramson Cancer Center, University of Pennsylvania, Philadelphia, PA 19104 USA; 7 Department of Pathology, University of Texas Southwestern Medical Center, Dallas, TX 75390 USA; 8 Department of Neuroscience and Physiology, State University of New York, Upstate Medical University, Syracuse, NY 13210 USA; 9 Department of Neurosurgery, State University of New York, Upstate Medical University, Syracuse, NY 13210 USA; 10 Department of Pediatrics, Icahn School of Medicine at Mount Sinai, New York, NY 10029 USA; 11 Department of Pathology, New York University Langone Health, New York, NY 10016 USA; 12 Simmons Comprehensive Cancer Center, University of Texas Southwestern Medical Center, Dallas, TX 75390 USA; 13 Children’s Medical Center Research Institute, University of Texas Southwestern Medical Center, Dallas, TX 75390 USA; 14 Department of Neurosurgery, University of Pittsburgh School of Medicine, 200 Lothrop St, Pittsburgh, PA 15213 USA; 15 Hillman Comprehensive Cancer Center, University of Pittsburgh Medical Center, 5115 Centre Ave, Pittsburgh, PA 15232 USA

**Keywords:** Glioma, Astrocytoma, Glioblastoma, Oligodendroglioma, Heterogeneity, Chromosomal instability, Microsatellite instability, Mismatch repair defects, Methylation profiling, Epigenetics

## Abstract

In recent years, the classification of adult-type diffuse gliomas has undergone a revolution, wherein specific molecular features now represent defining diagnostic criteria of IDH-wild-type glioblastomas, IDH-mutant astrocytomas, and IDH-mutant 1p/19q-codeleted oligodendrogliomas. With the introduction of the 2021 WHO CNS classification, additional molecular alterations are now integrated into the grading of these tumors, given equal weight to traditional histologic features. However, there remains a great deal of heterogeneity in patient outcome even within these established tumor subclassifications that is unexplained by currently codified molecular alterations, particularly in the IDH-mutant astrocytoma category. There is also significant intercellular genetic and epigenetic heterogeneity and plasticity with resulting phenotypic heterogeneity, making these tumors remarkably adaptable and robust, and presenting a significant barrier to the design of effective therapeutics. Herein, we review the mechanisms and consequences of genetic and epigenetic instability, including chromosomal instability (CIN), microsatellite instability (MSI)/mismatch repair (MMR) deficits, and epigenetic instability, in the underlying biology, tumorigenesis, and progression of IDH-mutant astrocytomas. We also discuss the contribution of recent high-resolution transcriptomics studies toward defining tumor heterogeneity with single-cell resolution. While intratumoral heterogeneity is a well-known feature of diffuse gliomas, the contribution of these various processes has only recently been considered as a potential driver of tumor aggressiveness. CIN has an independent, adverse effect on patient survival, similar to the effect of histologic grade and homozygous *CDKN2A* deletion, while MMR mutation is only associated with poor overall survival in univariate analysis but is highly correlated with higher histologic/molecular grade and other aggressive features. These forms of genomic instability, which may significantly affect the natural progression of these tumors, response to therapy, and ultimately clinical outcome for patients, are potentially measurable features which could aid in diagnosis, grading, prognosis, and development of personalized therapeutics.

## Introduction

As a group, diffusely infiltrating gliomas occur at a rate of 4.5 cases per 100,000 people in the United States annually (a mean of 16,800 cases) and comprise approximately 19% of all central nervous system (CNS) tumors [162, 163]. Originally described in 1865 by Rudolph Virchow, the diagnosis and classification of gliomas have undergone a number of revisions and updates in the intervening decades [56, 198]; however, until 2016, these diagnoses and tumor grades were based solely on histologic features [126]. In the 5th edition of the WHO Classification of Central Nervous System Tumors, released in 2021, adult-type diffuse gliomas were classified with a combination of histologic and molecular features as oligodendrogliomas (~ 7% of diffuse gliomas) in the presence of mutation in either *IDH1* or *IDH2* (hereafter grouped simply as “IDH mutation”) and co-deletion of chromosomal arms 1p and 19q, IDH-mutant astrocytoma (~ 11%) in the presence of an IDH mutation and retained 1p/19q, and IDH-wild-type glioblastoma (~ 82%) in the absence of either of these molecular alterations [127, 163]. IDH-mutant gliomas generally arise in the cerebral cortex with only rare exceptions in posterior fossa structures or spinal cord, in contrast to tumors defined by other drivers, including H3 K27M-mutant diffuse midline gliomas, and different progenitor cells may be more susceptible to malignant transformation by mutually exclusive oncogenic drivers [111, 199].

First identified as a molecular feature of diffusely infiltrating gliomas in 2008, IDH mutation confers a significantly better prognosis compared to their histologic grade-matched IDH-wild-type counterparts [127, 166, 231]. The majority of IDH mutations are *IDH1* R132H mutations, which can be identified with immunohistochemical stains [16, 22, 35, 36]. IDH mutation has been shown to be an early event in gliomagenesis [49, 168, 210], and results in the production of the oncometabolite 2-hydroxyglutarate (2-HG) [43, 52, 68, 210, 222, 230], which can be identified with magnetic resonance spectroscopy [9, 43]. The production of 2-HG inhibits α-ketoglutarate-dependent dioxygenases and induces widespread DNA hypermethylation, resulting in the glioma CpG island methylator phenotype (G-CIMP), causing significant transcriptional alterations, metabolic abnormalities, cellular dysregulation, and a poorly differentiated state [52, 69, 128, 139, 148, 160, 188, 189, 204, 210]. This methylation state can be used to distinguish IDH-mutant astrocytoma from other forms of diffusely infiltrating gliomas and other CNS neoplasms, and predict clinical outcomes [33, 34, 74, 108, 183, 227]. This global DNA methylation profiling also separates IDH-mutant astrocytomas into low- and high-grade clusters [33], as well as G-CIMP-low and G-CIMP-high clusters [38]. This G-CIMP-low subgroup has a lower level of methylation at some CpG sites and comprises a minority of IDH-mutant astrocytoma cases with significantly worse overall survival, relative to G-CIMP-high astrocytomas, although there is a general trend toward conversion from G-CIMP-high to -low with recurrence and progression in grade [38, 55, 121, 134].

In addition to diagnosis, additional molecular features have become increasingly important in tumor grading. The presence of *EGFR* amplification, *TERT* promoter mutation, and/or simultaneous gain of chromosome 7 and loss of chromosome 10 (+ 7/− 10) are currently considered molecular grade 4 features in IDH-wild-type glioblastoma, and homozygous loss of *CDKN2A/B* is considered a molecular grade 4 feature in IDH-mutant astrocytoma and a grade 3 feature in oligodendroglioma [127], although rare exceptions have been reported [11, 211]. A number of other genetic alterations have also been considered for diagnostic and grading schemes, including hemizygous *CDKN2A/B* loss and *CDKN2A/B* mutations [91, 110, 229, 234], as well as a number of other molecular features [10, 46, 72, 149, 177, 190, 224]. Unlike oligodendrogliomas and glioblastomas, which both frequently feature mutations in the promoter region of *TERT* to maintain telomere length, IDH-mutant astrocytomas frequently have mutations in *ATRX*, promoting an alternative lengthening of telomere (ALT) phenotype [152, 235]. Another key molecular feature associated with the clinical course of diffuse gliomas is *O*^*6*^*-methylguanine-DNA methyltransferase* (*MGMT*), which encodes a DNA repair protein that removes methyl groups from guanine residues caused by the administration of temozolomide (TMZ), the most common chemotherapeutic agent used in the treatment of malignant gliomas [79, 90, 156]. When the promoter region of *MGMT* is hypermethylated, however, the cellular level of MGMT protein is decreased, leaving the effects of TMZ unchallenged, leading to tumor cell death via intact mismatch repair (MMR) mechanisms [64, 71, 213].

Another factor, imperative to understanding the pathogenesis of these tumors, is intratumoral heterogeneity, which has been definitively shown in recent years with single-cell sequencing techniques and geographically targeted biopsies/autopsy specimens, and recapitulated in organoid models [2, 4, 80, 95, 129, 133, 147, 167, 207, 214, 237]. As a general rule, diffuse gliomas are composed of numerous subclones comprised of cells with different epigenetically regulated transcriptional states, different mutational or copy number states, and different microenvironmental states and interactions [156, 201]. This principle becomes more complex with tumor recurrence and treatment, and even genetic alterations thought to be foundational to tumorigenesis, such as IDH mutation, may become subclonal and relegated to “passenger mutation” status with tumor evolution, while other newly acquired mutations may emerge in dominant clones and drive tumor biology [17, 65, 100, 132, 138, 142, 215]. This heterogeneity makes diffusely infiltrating gliomas incredibly diverse and adaptable and is believed to contribute significantly to resistance to immune regulation and therapeutics.

**Fig. 1:**
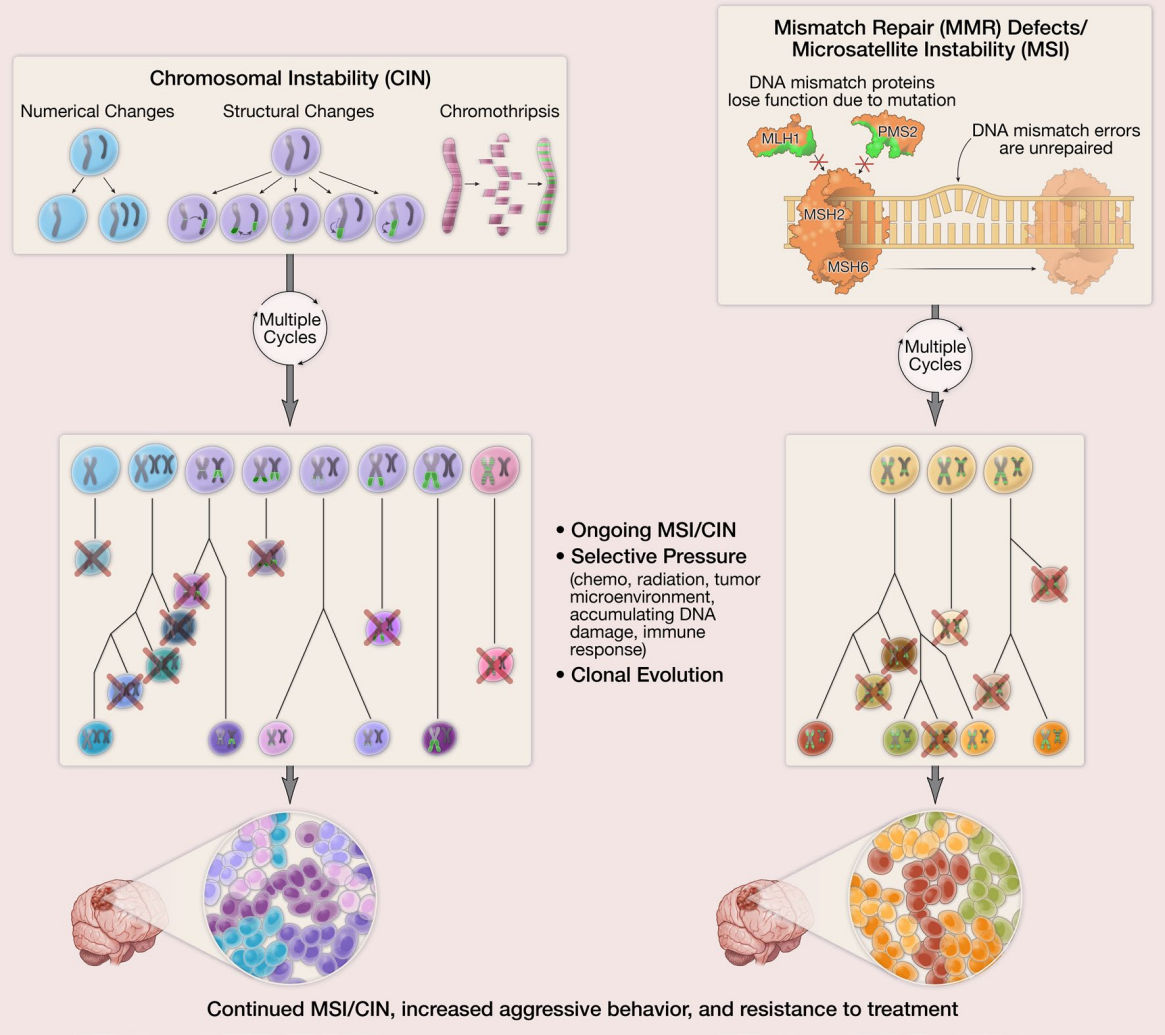
Overview of the mechanisms and consequences of chromosomal instability (CIN) and microsatellite instability (MSI)/mismatch repair (MMR) mutations and the resulting intratumoral heterogeneity. Figure created by Melissa Logies

Herein, we review processes that contribute to the rapid development of intercellular heterogeneity, including forms of epigenetic instability, chromosomal instability, and mismatch repair (MMR) defects/microsatellite instability (MSI). These processes are well-known to drive cancer formation and progression in a wide variety of cancer types through production of a population of tumor cells with increased genetic and phenotypic diversity, followed by Darwinian selection of the tumor clones most suited to survival, invasion, progression, metastasis, and resistance to therapy (Fig. 1), but their contribution to gliomagenesis and malignant progression of gliomas has only recently been uncovered. Genetic instability in its various forms has been identified in all subtypes of adult-type diffusely infiltrating gliomas, including IDH-wild-type glioblastoma, IDH-mutant astrocytoma, and oligodendroglioma. In the IDH-mutant astrocytoma category, in particular, there is evidence that these processes significantly impact biologic behavior [17, 175, 179, 209], suggesting that forms of genetic and epigenetic instability may be key to understanding the natural history of IDH-mutant astrocytoma, and may have significant prognostic implications as well as the potential to serve as therapeutic targets.

## Defining tumor heterogeneity using single-cell transcriptomics

In the past decade, single-cell sequencing technology has revolutionized the way we look at cancer, providing extraordinary granularity of intratumoral and intertumoral heterogeneity as it relates to cellular diversity. A notable contribution of this technology has been toward the deconvolution of cellular states linked to developmental lineages or molecular processes and the discovery of rare cell types. Single-cell transcriptomics (scRNA-seq) and open chromatin accessibility (scATAC-seq) have been the two most widely used -omics modalities thus far, enabling unprecedented dissection of tumor composition and functional state, in both IDH-wild-type and IDH-mutant diffuse gliomas. In contrast, single-cell DNA-seq has been more technically challenging and thus less informative of single-cell level resolution mutational heterogeneity and clonal evolution in gliomas [207]. The earliest scRNA-seq glioma studies elegantly demonstrated the power of this technology to resolve tumor heterogeneity in GBM, as well as to infer specific copy number variations (CNVs) using gene co-expression data [167]. Subsequent transcriptomic studies have focused on consolidating GBM heterogeneity into distinct but shared cellular states [77, 155, 218, 219, 236], including developmentally coopted hierarchies that recapitulate gliogenesis [50, 170].

Profiling of IDH-mutant gliomas using single-cell sequencing has demonstrated a more conserved tripartite tumor hierarchy with less overall heterogeneity, compared to IDH-wild-type GBM. In grade 2 IDH-mutant oligodendrogliomas, scRNA-seq defined a developmental hierarchy in tumor cells, with a small population of stem-like proliferative glioma cells resembling neural stem/progenitor biology at one apex along with two differentiation trajectories, one towards mature oligodendrocyte-like and the other towards mature astrocyte-like apices [208]. A similar hierarchy was reconstructed in IDH-mutant astrocytomas in a follow-up study, suggesting shared developmental lineages between these otherwise genetically distinct IDH-mutant gliomas [214]. Interestingly, the proportion of stem-like malignant cells expanded with higher grade, in both IDH-mutant astrocytoma and oligodendroglioma [214]. As well, the “oligodendrocyte-like” differentiation state was shown to be “stalled” with an aberrantly blocked myelination transcriptional program [223]. Such analyses have been enhanced by longitudinal studies in primary/recurrent IDH-mutant gliomas specimens, tracking changes and stability in persistent and recurrent tumor subpopulations [21]. Additionally, multi-omic analyses, including scRNA-seq plus scATAC-seq [3, 12, 223] or scRNA-seq plus single-cell DNA methylome [39, 103], have enhanced transcriptional cell state diversity analysis and helped to reconstruct more accurate transcription factor regulatory networks. Single-cell transcriptomics has informed heterogeneity not only in tumor cells but also in the surrounding infiltrated non-neoplastic parenchyma, which includes microglia/macrophages, oligodendroglial-lineage cells, astrocytes, and neurons, collectively called the tumor microenvironment (TME) [154]. Important for GBM, it was shown that macrophage–tumor cell interaction not only drives MES transition in tumor cells but in macrophages themselves [41, 89]. The most notable differences observed so far between IDH-mutant and IDH-wild-type high-grade gliomas relate to their myeloid TME composition and functional properties [1, 70, 169]. With the exciting emergence of IDH inhibitors showing therapeutic efficacy in early clinical trials [144–146], this technology can be used to probe important mechanistic and translational questions related to treatment response and resistance, with the first of such studies providing an early glimpse into the biology of response and resistance in human IDH-mutant oligodendrogliomas after treatment with vorasidenib [194].

**Fig. 2:**
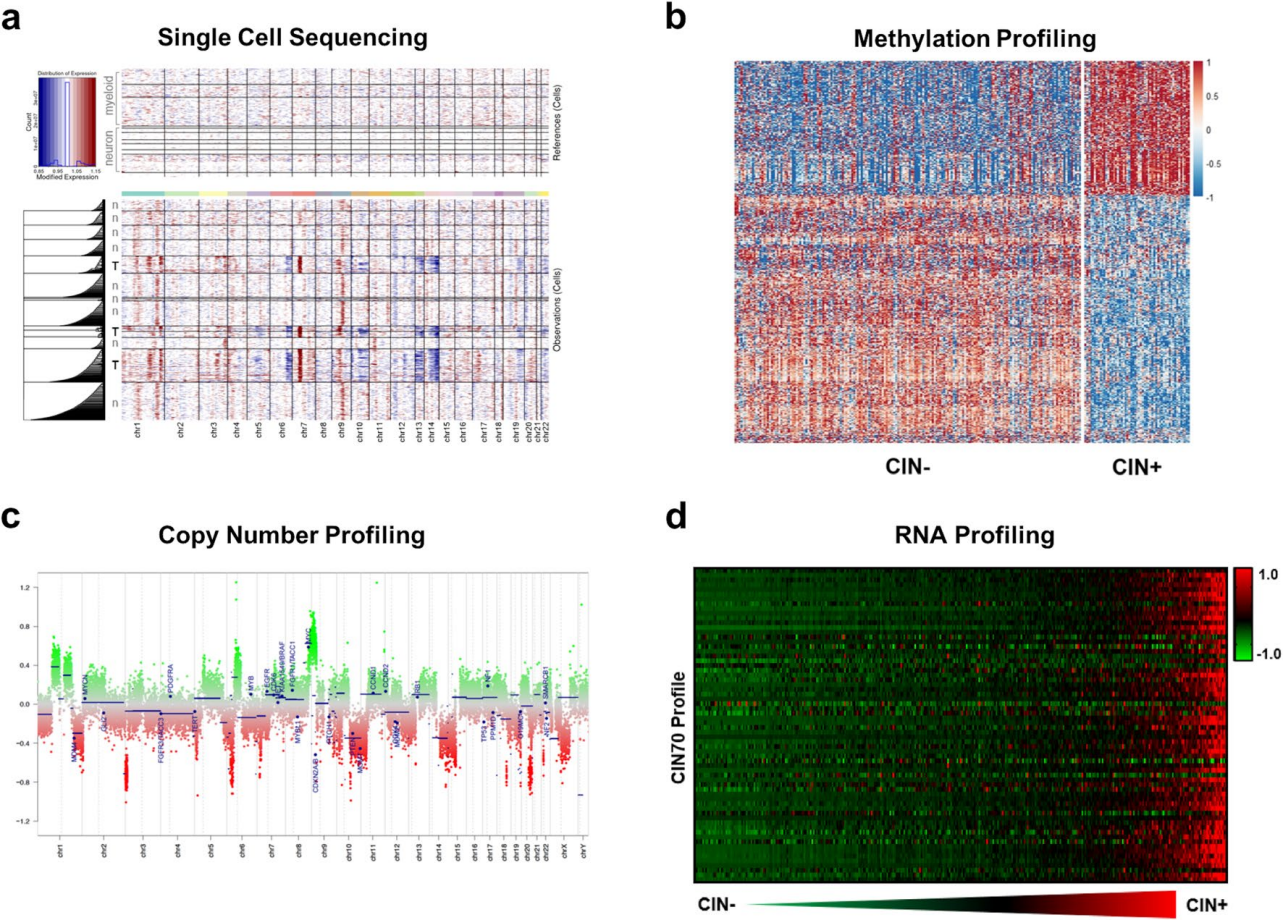
Measurement of chromosomal instability in IDH-mutant astrocytomas. Chromosomal instability can be identified via **a** single-cell sequencing, **b** methylation profiling, **c** identification of incongruous “chromosomal complexity” or high levels of copy number alterations spread throughout the genome, and **d** mRNA profiling. Panel c is adapted from Lyon et al., 2021 [133], and is used with permission under Creative Commons Attribution 4.0 International Licenses (http://creativecommons.org/licenses/by/4.0/)

While the use of single-cell DNA sequencing to characterize mutations has been technically challenging, scRNA-seq and scATAC-seq enable the inference of genetic CNVs using several different computational pipelines [76, 153, 157, 167]. Inference of CNVs allows for the accurate annotation of tumor cell identity in scRNA-seq data. Furthermore, it has enabled us to define CNV heterogeneity within different tumor niches in IDH-mutant and IDH-wild-type high-grade gliomas, including between necrotic tumor core and infiltrative tumor margin (Fig. 2a). It represents an adaptable, orthogonal method to explore still unanswered questions in the field, related to clonal tumor evolution and glioma cell-of-origin. With the emergence of user-friendly bioinformatic toolsets, reduced cost, and the ability to perform single-cell sequencing and spatial transcriptomics on FFPE samples, this powerful technology will likely become a diagnostic companion to every neuropathologist in the near future.

## Chromosomal instability (CIN)

Chromosomal instability (CIN) is an ongoing process of relatively rapid chromosomal alterations resulting in numerical abnormalities (aneuploidy) and/or structural abnormalities, and may involve catastrophic, large-scale fragmentation and reassembly of one or more chromosomes in a single event (a process known as chromothripsis), as well as unequal segregation on extrachromosomal DNA (ecDNA) [48, 57, 130, 159, 191, 193, 196, 216]. CIN is a continuing process that can lead to the rapid loss or alteration of tumor suppressor genes, a gain of oncogenes, disruption of genetic and epigenetic architecture, and/or formation of gene fusions, followed by clonal evolution (Fig. 1) in the face of cellular stressors, which may include clonal competition due to resource availability, tumor microenvironment, immune system interactions, and therapeutic challenges [17, 102, 156, 217]. Recent work has shown that individual cells isolated from cancers with proven chromosomal instability may fully recapitulate the intercellular heterogeneity found in the parental population within approximately 22 generations [86]. It is important to stress that although CIN frequently results in aneuploidy, these two terms are not synonymous, and the presence of aneuploidy is not necessarily evidence of CIN [8]. For example, oligodendrogliomas are defined by co-deletion of chromosomal arms 1p and 19q (in addition to IDH mutation), but this represents a form of *stable* aneuploidy, mediated by an unbalanced translocation between chromosomes 1 and 19, with subsequent loss of the derivative chromosomal fragment der(1;19) (q10;p10) [82, 98, 171].

CIN has been well-described in several cancers, perhaps most famously in colorectal cancer, where mutations in the *APC* gene result in dysregulation of WNT signaling as well as interference with microtubule function during mitosis, leading to a CIN phenotype [29, 81, 158]. Numerous cancers and syndromes have CIN as a key component, including multiple carcinoma types, hematologic malignancies, and Fanconi’s anemia [13, 42, 44, 60, 117, 185, 203], and recent work has suggested that approximately 80% of samples across a wide variety of human cancers have some detectable degree of CIN as a component of their disease process [60, 212]. Mutations in a number of genes with functions involving DNA/chromosome repair, mitotic control and fidelity (including centrosome function, spindle assembly, and mitotic fidelity/checkpoints), and apoptosis, among others, have been positively implicated in the development of chromosomal instability, although given the number of proteins involved in mitosis and cell replication, it has been hypothesized that loss of function in any one of as many as 2,000 genes could potentially result in some form of chromosomal instability [14, 30, 177, 197, 205, 206, 216].

Given that CIN is an ongoing process and pathologic specimens generally represent only a single point in time, detection of CIN may be difficult, and a number of potential direct and indirect methods of measurement have been proposed [118]. The most direct and conclusive method for identifying CIN in a tumor sample is cell culture where changes in karyotype and/or single-cell copy number alteration (CNA) in successive generations of cells can be directly observed; however, due to the time and costs associated with this method, this is generally not practical for routine clinical evaluation [86, 116, 117]. Indirect methods to infer CIN include evaluating histologic features such as anaphase segregation errors (including chromatin bridges and lagging chromosomes), double minute/circular extrachromosomal DNA (ecDNA), and micronucleus formation [19, 20, 57, 159, 181, 195, 232]. Other techniques involve the assessment of intercellular variation in aneuploidy with comparative genomic hybridization (CGH) [118], fluorescent in situ hybridization (FISH) [44, 185, 203], and single-cell sequencing (Fig. 2a) with inferred CNA levels between cells [147, 167, 237]. Additional methods in IDH-mutant astrocytomas and other neoplasm types have involved global DNA methylation signatures (Fig. 2b) [114, 125], CNA profiling to identify “chromosomal complexity”/“copy number heterogeneity” (Fig. 2c) [150, 173, 174, 176, 228], mRNA expression signature patterns (Fig. 2d) [37, 175], and other computational molecular signatures of CIN [60].

In diffuse gliomas, the impact of CIN is less clear than in other systemic cancers. Multiple recent studies have shown that IDH-mutant astrocytomas accumulate increasing CNA with recurrence [133, 177], as they progress from a mean CNA level of 9.3 ± 0.5% (~ 290 Megabase pairs [Mbp]) in CNS WHO grade 2 to 20.9 ± 1.8% (~ 650 Mbp) in CNS WHO grade 4 [48, 173–177, 190], and/or with the development of defined high-grade molecular features, such as homozygous *CDKN2A* loss [150]. This accumulation of chromosomal complexity occurs in a much more pronounced manner compared to glioblastoma [175] or oligodendroglioma [178], and unlike oligodendroglioma or glioblastoma, this total/overall CNA level (a snapshot of the level of genomic disruption at the time of surgery) tends to be scattered across the genome with relatively few consistent recurrent sites of alteration [73, 150, 174, 176, 212, 228]. While not a definite signal of CIN, this progressive rise in CNA in parallel with tumor progression is indicative of an ongoing process of genome alteration with accumulating gains and losses.

Previous studies in large cohorts of IDH-mutant astrocytomas and IDH-wild-type glioblastomas have generated several lines of evidence suggesting that elevated CNA levels and CIN may be present early in the development of a select subset of tumors (prior to developing high-grade histologic or molecular hallmarks), and that this finding has predictive power, identifying glioma subsets with more aggressive behavior and worse clinical outcomes in terms of the patient’s recurrence-/progression-free survival and overall survival. While overall CNA levels tend to be significantly elevated in cases with either grade 4 histology or equivalent molecular features [150, 175], there is a subset of grade 2–3 IDH-mutant astrocytomas (approximately 15% of cases) in which incongruously elevated overall CNA levels are associated with dismal survival (progression-free survival (PFS) ≤ 12 months and overall survival ≤ 24 months), and this is true when selecting specifically for elevated overall CNA or poor clinical outcomes and excluding cases with other known high-grade molecular features [174–176]. We have shown that overall CNA level at the time of initial surgery is an independent prognostic factor in IDH-mutant astrocytoma, in which cases with CNA comprising ≥ 15% of the total genome (approximately 465 Mbp) have significantly worse Karnofsky performance status, progression-free survival, and overall survival compared to their counterparts with relatively low CNA [125, 150, 151, 174–176], a finding which was validated in other glioma cohorts and other cancers, although the particular CNA threshold varied somewhat [10, 190, 212]. IDH-wild-type glioblastomas followed a similar trend; the vast majority of cases had high overall copy number variation, but the few cases with < 10% total copy number variation had a significantly better clinical outcome in terms of both progression-free and overall survival [172, 175, 212].

Other data have shown that patients with IDH-mutant astrocytomas harboring mutations in genes known to be involved with maintenance of chromosomal stability in other cancer types (*APC, BLM*, *BRCA1/2*, the Fanconi anemia family genes, among numerous others) have higher overall CNA at the time of initial surgery and correspondingly poor clinical outcomes regardless of histologic grade [150, 174, 175, 177]. Cases with mRNA expression patterns and global DNA methylation patterns associated with known CIN had higher overall CNA levels and worse clinical outcomes compared to cases with mRNA and methylation patterns consistent with chromosomal stability, in terms of progression-free survival (median PFS of 29–40 months vs. 70–88 months, respectively) and overall survival (median OS of 41–63 months vs. 87–140 months, respectively) [37, 125, 175, 177]. In addition, genetic analysis of multiple biopsies, resections, and autopsy specimens within individual patients with diffuse glioma has demonstrated significant regional heterogeneity of chromosomal alterations and heterogeneity of specific epigenetic alterations (including *MGMT* promoter methylation), as well as mounting overall CNA levels in the recurrent tumor samples, an effect that was more pronounced and was more predictive of clinical behavior in IDH-mutant tumors than other glioma types, although this may be driven in part by treatment, including radiotherapy [133]. There have been several studies utilizing single-cell sequencing technology to investigate genomic heterogeneity in multiple types of diffusely infiltrating gliomas; however, these studies have generally had a relatively small number of included tumors and have not been focused on uncovering CIN, although inferred CNA analysis in these studies demonstrates copy number profile heterogeneity in some cases [147, 167, 207, 237].

## Mismatch repair (MMR) protein defects and microsatellite instability (MSI)

The mismatch repair (MMR) system is an evolutionarily conserved system comprised in humans of the paired proteins, MSH2–MSH6, which recognize base-pair mutations and mismatches, and then recruit the MLH1–PMS2 complex, as well as DNA ligase I, DNA polymerase (POLD1/POLE), PCNA, and EXO1 (among other proteins) to excise and repair incorrect base pairing, ensuring fidelity of DNA replication [99, 120, 137]. Inactivation of the genes involved in this process (primarily *MSH2*, *MSH6*, *MLH1*, and *PMS2*) predictably results in an increased rate of somatic mutations, high tumor mutational burden (TMB; termed “hypermutation” when there are ≥ 10 mutations/Mb), and microsatellite instability [187]. Microsatellite instability is a consequence of MMR defects and occurs with an increased rate of mutation in repeated sequences of DNA (microsatellites) across the genome [192], as well as an increased rate of mutations affecting proto-oncogenes and tumor suppressors with subsequent clonal evolution, similar to that seen in CIN (Fig. 1) [17, 102, 156, 209, 217]. Mutation, deletion, or promoter methylation of these MMR genes is present in approximately 12% of ovarian cancers, 15% of colorectal cancer, 22% of gastric cancers, and up to 30% of endometrial cancers, as well as heritable cancer syndromes, such as Lynch syndrome (monoallelic germline mutation of an MMR gene) and constitutional mismatch repair deficiency (CMMRD; biallelic germline mutation of an MMR gene), which have an increased risk of numerous cancers [40, 84, 105, 106]. In diffusely infiltrating gliomas, mutations in MMR genes may occur as part of a germline syndrome [28, 59, 75, 84, 105, 106, 161, 200], as sporadic mutations [27, 32, 179, 209], or as the result of temozolomide therapy, either due to the selection of temozolomide-resistant MMR-deficient clones or by temozolomide-induced mutation in one of the MMR genes [6, 17, 31, 45, 53, 94, 104, 179, 209, 213, 233].

A recent case series by Suwala et al. described a distinct subtype of IDH-mutant astrocytoma with MMR mutations occurring in the setting of Lynch syndrome or CMMRD, termed primary mismatch repair-deficient IDH-mutant astrocytoma (PMMRDIA) [200]. These tumors occurred primarily in children and young adults (although this cohort included patients up to 54 years), had a hypermutant phenotype and microsatellite instability, high-grade morphology, low rates of *MGMT* promoter methylation, and dismal prognosis with a median survival of only 15 months. Interestingly, these tumors were epigenetically distinct from IDH-mutant astrocytomas, with reduced global methylation levels, and were distinguishable with methylation profiling from secondary MMR-deficient tumors, which tended to match better with their MMR-retained IDH-mutant astrocytoma counterparts [59, 200]. This may also be true of IDH-wild-type glioblastomas [87], suggesting that there may be fundamental differences in the biology of tumors with acquired and germline MMR mutations.

While sporadic MMR mutations are relatively rare in diffuse gliomas, those occurring in recurrent gliomas in the setting of TMZ administration are much more frequent [17, 27, 32, 38, 179, 209], and appear to be more common for IDH-mutant astrocytomas (47%) than IDH-wild-type glioblastomas (16%) or oligodendrogliomas (25%) [17, 94]. MMR mutations occurring after TMZ administration may occur either through the selection of MMR-deficient subclones already present in the heterogeneous tumor cell population or by TMZ directly inducing mutation in an MMR gene, particularly in tumor cells with methylated MGMT [17, 31, 45, 53, 94, 102, 107, 179, 209, 213, 217, 233]. These MMR mutations precede TMB increase [17, 179, 209], and subsequent clonal expansion of the surviving MMR-deficient subclone may then contribute to further TMZ resistance [51, 209]. However, this TMZ resistance may potentially be countered with poly(ADP-ribose) polymerase inhibitors (PARPi) [92], inhibitors of the RecQ DNA helicase WRN [18, 40, 122], and newer chemotherapeutic agents specifically designed to induce DNA damage and death of MGMT-deficient tumor cells in an MMR-independent manner [123].

**Fig. 3:**
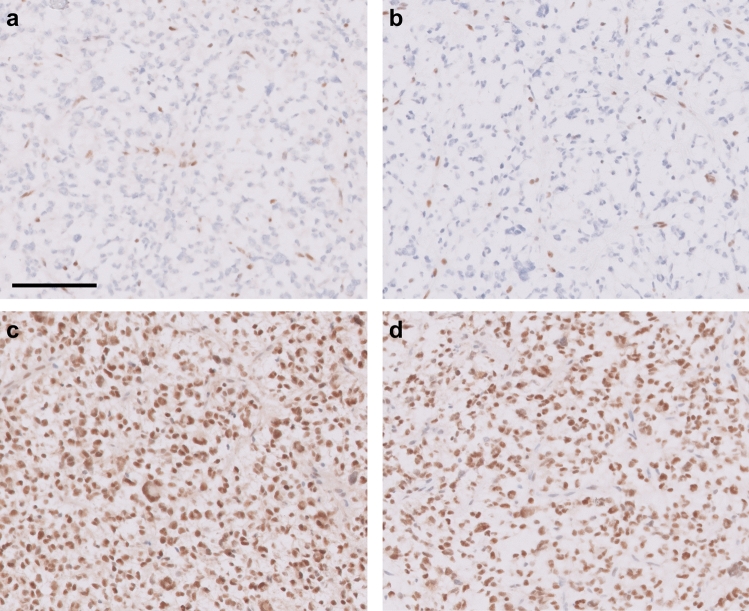
Mismatch repair protein deficits. Photomicrographs demonstrate loss of **a** MSH2 and **b** MSH6 expression with retention of both proteins in non-neoplastic background cells and retention of **c** PMS2 and **d** MLH1 in tumor cells. All images are taken at ×200 total magnification, scale bar = 100 µm

MMR mutations can be detected through routine NGS panels or immunohistochemistry (IHC) with loss of staining for one or more MMR proteins (Fig. 3) [7, 28, 140, 141, 179, 200]. IHC may be particularly useful as non-neoplastic brain cells (native glial cells, neurons, and endothelial cells) retain nuclear reactivity in cases of acquired MMR mutation, while these cells should also be negative in cases with germline mutations [141, 179, 200], although notably in many cases mutations resulting in MMR-deficiency are only present at a subclonal level, which may be evident with IHC staining [140, 209]. No matter the mechanism of loss of MMR function, these tumors often have distinct and striking morphology with diverse cellular appearances including areas with primitive neuronal component, ependymoma-like areas with perivascular pseudorosettes, large bizarre nuclei, multinucleate giant cells, and frequent atypical mitotic figures including Creutzfeldt-like cells and tripolar mitoses [84, 105, 140, 179, 200].

MMR-mutation and the resulting hypermutant phenotype have been associated with worse clinical outcomes in terms of more frequent and rapid recurrences, tumor progression, and patient death in IDH-mutant astrocytomas compared to grade-matched counterparts in many, but not all studies [17, 124, 179, 200, 209]. Some authors have suggested that germline MMR mutations may be sufficient to warrant WHO grade 4 status, regardless of other histologic or molecular features [200]. Whether they are inherited or acquired, these mutations are frequently associated with high-grade histologic and molecular features and are often associated with progression in grade when acquired between sampling, but they may confer poor prognosis even when identified in IDH-mutant astrocytomas with no otherwise worrisome features, suggesting they may serve as a useful biomarker in certain circumstances [179, 200, 209]. MMR mutations also increase the number of neoantigens (mutant proteins potentially viewed as novel by the immune system) [15, 109, 112, 135], with some data suggesting that as many as 42% of nonsynonymous mutations in exon regions may result in neoantigen formation [17]. This property makes immune checkpoint inhibitor therapy an attractive adjuvant treatment option for these tumors [24, 47, 53, 83, 87, 93, 101, 112, 131, 180, 182, 209], although not all hypermutant cancers respond [112, 143, 180, 209]. Other studies have suggested that immune therapy may only remove MMR-mutant subclones [140, 180, 220], so this therapy may be most effective in patients with germline mutations as the MMR mutations are more uniform in these cases [24, 200].

## Epigenetic plasticity, epigenetic instability, the G-CIMP phenotype, and *MGMT* promoter methylation

Conceptually, epigenetics involves the control of and changes to gene function and expression that does not involve changes to the actual DNA sequence, including modifications such as acetylation and methylation of DNA and histones, which result in the activation, silencing, and other modulation of gene expression [62]. In part, epigenetic alterations control the “fate” of the cell under normal conditions; all cells in an organism theoretically contain the same DNA content, however, the epigenetically driven gene expression pattern is thought to result in cellular differentiation from stem cells [62]. Many tumor types, including gliomas, are thought to contain cancer stem cells, which retain a more dedifferentiated state as well as multiple clones of differentiated cells with phenotypic variation and plasticity, which may include features favorable to tumor cell survival, including ability to evade the immune system, resistance to therapy and hypoxia, better ability to infiltrate the surrounding neuropil, and advantageous metabolic states [66, 67, 156]. As described above, IDH mutation and the resulting production of the oncometabolite 2-HG results in a global hypermethylated state in IDH-mutant astrocytomas, which can be detected with DNA methylation profiling and can be used to discriminate these tumors from other histologically similar neoplasms, including oligodendroglioma and glioblastoma [33]. The G-CIMP methylation phenotype can further be distinguished into relatively highly methylated (G-CIMP-high) and lowly methylated (G-CIMP-low) groups [38, 55, 134]. The vast majority of IDH-mutant astrocytomas are classified as G-CIMP-high, and have a superior prognosis to the G-CIMP-low subgroup, which accounts for only 6–17% of primary and recurrent tumors, and is characterized by more frequent CNA and chromothripsis, a more stem-cell-like molecular signature, and epigenetic features similar to IDH-wild-type glioblastoma [38, 56, 63, 150, 175].

Primary IDH-mutant astrocytomas are predominantly lower grade (2021 CNS WHO grades 2–3) and are predominantly classified as G-CIMP-high, an epigenetic phenotype retained in ~ 70% of recurrent cases. There is also an intermediate G-CIMP subgroup in recurrent cases, representing those which are slowly evolving from low to high grade, and trending from G-CIMP-high to G-CIMP-low ("epigenetic plasticity”), which is associated with malignant progression [55], similar in concept to rising CNA levels with increasing grades of IDH-mutant astrocytoma [175]. There was also a small “epigenetically unstable” subgroup that converted rapidly from G-CIMP-high to G-CIMP-low status with recurrence that exhibited significantly worse clinical outcomes (with a predictive panel of 7 CpG sites) [55], similar to incongruously high CNA in low-grade IDH-mutant astrocytomas with rapid recurrence and patient death [10, 151, 172, 174, 176, 190]. Notably, a portion of IDH-mutant astrocytomas, including tumors which were designated WHO grade 4 at initial presentation, were relatively stable; ~ 35% maintained a G-CIMP-high status and ~ 15% moved to an intermediate epigenetic phenotype, suggesting that this progression is related to but not completely dependent upon grade [55].

**Fig. 4:**
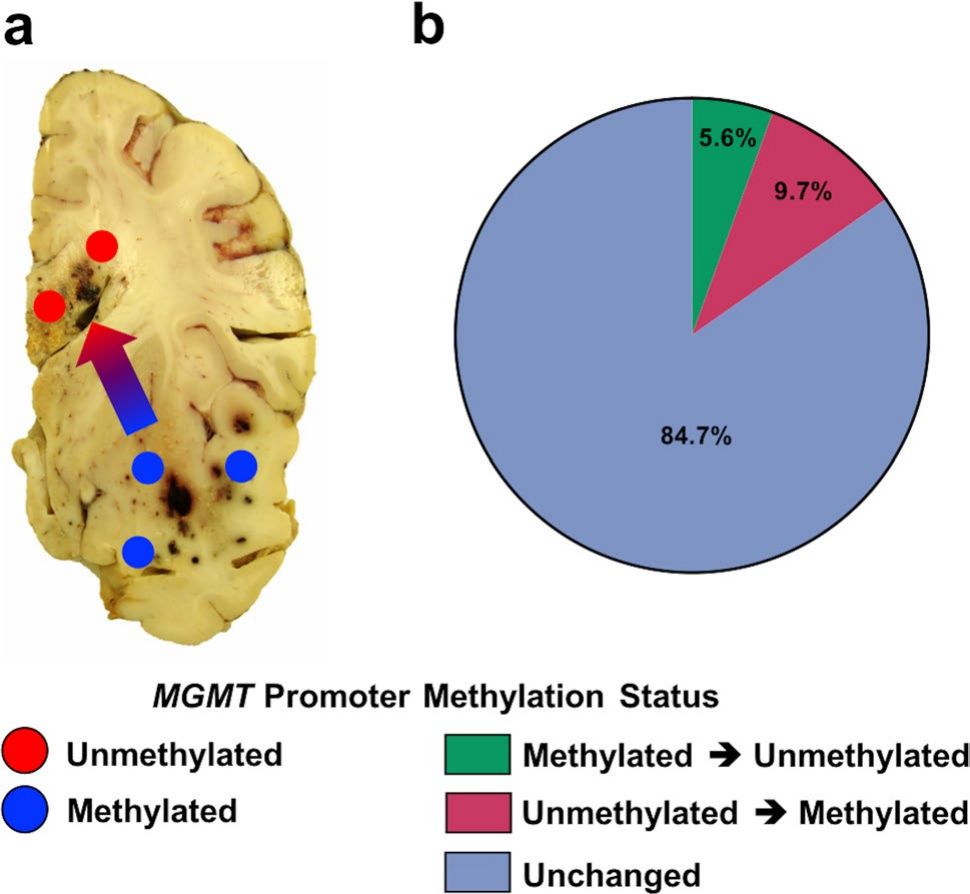
Spatial and temporal *MGMT* promoter methylation status change. **a** Case study demonstrating *MGMT* promoter methylation at the site of initial biopsy in the right temporal lobe and immediately adjacent tumor at autopsy with loss of *MGMT* promoter methylation in more distant brain regions in the right medial frontal lobe and corpus callosum [133], and **b** relative frequency of change in *MGMT* promoter methylation status in initial and recurrent diffuse glioma samples [17, 133, 173, 175, 176]

Another important epigenetic marker associated with treatment and outcome in IDH-mutant astrocytoma is *MGMT* promoter methylation [79, 90]. Although many neuropathology practices do not routinely re-test *MGMT* promoter methylation at recurrence or in multiple regions during surgery, recent reports have indicated that among other genetic and epigenetic changes, there may be spatial and/or temporal differences in *MGMT* promoter methylation status (Fig. 4a), particularly after treatment with TMZ [17, 23, 58, 61, 78, 133, 156, 165, 225]. There may be significant epigenetic heterogeneity at this particular locus, and progression from hypermethylated to hypomethylated *MGMT* promoter status in recurrent tumors may reflect the selection of existing hypomethylated *MGMT* cells and serve as a mechanism for further resistance to TMZ [25, 156]. Analysis of pooled cohorts of diffusely infiltrating gliomas with paired *MGMT* promoter status evaluation in both paired primary and recurrent tumors [17, 38, 104, 133] demonstrates that ~ 15% of cases have discrepancies in *MGMT* promoter methylation status and may either develop or lose promoter methylation at the time of recurrence (Fig. 4b), although the available sample size is too small to make definitive conclusions on the prognostic or treatment effects of this change.

Other recent work has suggested a possible mechanism for epigenetic instability. During DNA replication, epigenetic modifications, including histone methylation, must be preserved in the daughter DNA strands [184]; this process is complex but broadly is accomplished by the distribution of recycled parental histones split between two daughter copies with the synthesis of new histones in an approximately 50:50 ratio on each chromosome and subsequent duplication of epigenetic modifications onto the newly synthesized histones [88, 136, 184]. This process is complicated by a number of factors, including the availability of nucleotides, DNA damage, and formation of secondary DNA structures (such as guanine-rich quadruplexes, which may be crucial to genetic instability with *ATRX* inactivation) [54, 115, 119, 164, 184, 202, 221], which pause replication and histone recycling/incorporation [96, 97]. When histone recycling is then disconnected from DNA replication these regions are populated primarily by newly synthesized histones which lack the epigenetic modification of the parental DNA and, as a result, will tend toward loss of methylation in these particular regions [184]. The failure of this process and the resulting loss of fidelity in histone methylation becomes more likely with significant DNA damage, including that associated with CIN and MSI. A number of genes are known to be specifically associated with this process, and mutations in *BLM*, *FANCJ*, *PRIMPOL*, *REV1*, and *WRN*, among others, have been associated with this form of epigenetic instability [119, 184, 186]. Interestingly, mutations in the first four of these genes were identified in cases with loss of *MGMT* promoter methylation in recurrences (Fig. 4b), suggesting that this process may be related to changing *MGMT* promoter methylation in at least some cases.

## Prognostic and therapeutic implications of instability and heterogeneity

**Fig. 5:**
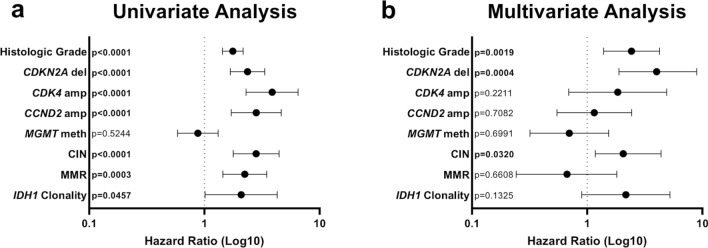
Forest plots of Cox proportional hazard regression analysis. **a** Univariate and** b** multivariate analysis of hazard ratios (HR) and 95% confidence intervals (CI) of features in IDH-mutant astrocytomas performed on three distinct publicly available cohorts (*n* = 518) [17, 38, 104]. In the multivariate model, histologic grade, homozygous *CDKN2A* deletion, and chromosomal instability are independently associated with significantly worse clinical outcomes. Individual molecular features were not available for all cases; analysis was performed using MedCalc Statistical Software version 22.007 (MedCalc Software Ltd, Ostend, Belgium)

Numerous studies on the various forms of genetic and epigenetic instability in IDH-mutant astrocytomas (and other diffusely infiltrating gliomas and CNS neoplasms) have yielded somewhat mixed results. In general, however, these molecular mechanisms tend toward increasing intercellular heterogeneity, which appears to drive progression and aggressiveness in these tumors after Darwinian selection of clones with increased survival potential, including resistance to therapy and immune regulation, similar to the effect of these mechanisms in other systemic cancer types [205, 216]. Univariate analysis, performed on three combined cohorts of publicly available IDH-mutant astrocytoma cases (*n* = 518) [17, 38, 104], demonstrated a significantly detrimental effect of increasing histologic grade (*p* < 0.0001), homozygous *CDKN2A* deletion (*p* < 0.0001), *CDK4* amplification (*p* < 0.0001), *CCND2* amplification (*p* < 0.0001), chromosomal instability (*p* < 0.0001), MMR mutation (*p* = 0.0003), and subclonality of *IDH1* mutation (*p* = 0.0457), while *MGMT* promoter methylation was not significantly associated with patient survival (Fig. 5a). In multivariate analysis, histologic grade (*p* = 0.0019), homozygous CDKN2A deletion (*p* = 0.0004), and CIN (*p* = 0.0320) were significantly associated with impaired survival in IDH-mutant astrocytoma patients, suggesting that these features are *independently* associated with poor outcomes in this diffuse glioma subset, while factors such as *MGMT* methylation status, MMR mutation, and IDH1 clonality were not (Fig. 5b). Although the presence of MMR mutation was not independently associated with poor outcome in multivariate analysis, these mutations were significantly correlated with higher initial histologic and molecular grade as well as progression to WHO grade 4 [179], and some authors have suggested that some instances of MMR mutation, particularly cases with germline mutation, may warrant an automatic WHO grade 4 designation [200]. Similarly, CIN was considered for inclusion as a molecular prognostic factor by The Consortium to Inform Molecular and Practical Approaches to CNS Tumor Taxonomy–Not Official WHO (cIMPACT-NOW) in 2020; however, it was decided at the time that more evidence was needed as well as consensus on a threshold of CNA [26]. As technology that can readily detect these processes becomes more widespread, future WHO grading recommendations may be revised to include a more granular assessment of these genetic and epigenetic processes to further refine astrocytoma prognosis. Leveraging single-cell transcriptomics technologies to infer copy number alterations with single-cell resolution represents one such promising endeavor to better define the CIN landscape with high granularity and spatial resolution (Fig. 2a).

In addition to prognostic and predictive importance, the identification of these instability patterns may provide a significant therapeutic opportunity [235]. Given that the hypermutator phenotype that frequently accompanies MMR mutation produces numerous neoantigens [109, 112, 135], some studies have found a benefit in treatment with immune checkpoint inhibitors, although the evidence is mixed, and these therapies may only affect hypermutant subclones with MMR-mutations [17, 24, 93, 101, 140, 182, 209, 220]. While CIN has been associated with resistance to traditional therapeutic strategies [113], dozens of drugs are currently in clinical trials or have been previously approved by the Food and Drug Administration (FDA) for other cancer types aimed at disrupting the process of CIN or further inducing CIN to lethal levels [177, 193, 205]. Compounds aimed at reducing CIN generally promote mitotic checkpoints and inhibit cell division, while therapies which enhance CIN further destabilize the cell replication process (microtubule and centrosome dynamics, mitotic kinases, mitotic checkpoints, chromatin modification, etc.) to push DNA damage past a viability threshold in cells that are already prone to developing CIN, although concerns remain for the potential of this strategy to promote more malignant and aggressive tumor cell clones [193, 205].

## Conclusions

Tumorigenesis and molecular evolution in adult-type diffusely infiltrating gliomas, including IDH-mutant astrocytomas, is a complex process that involves widespread genetic and epigenetic changes that occur in highly complex and evolving tumor microenvironments. While gliomas are generally defined and classified according to a small number of discrete molecular alterations, the genetic landscape is far more complicated, with numerous detrimental alterations affecting a wide range of fundamental cellular processes, and there may be significant cell-to-cell heterogeneity. As these tumors are widely considered to be surgically incurable and standard chemotherapy and radiation therapy ultimately have not proven successful at curing the majority of cases, future rational treatment of these tumors must resolve these issues and address heterogeneity both in neoplastic cells as well as in the non-neoplastic tumor microenvironments in which these tumors emerge and grow if it has any hope of success [5, 85, 104, 156, 188, 226, 235]. In addition, the processes of genetic and epigenetic instability outlined here should be studied further in large cohorts to determine more precise prognostic implications and how this ongoing process of DNA and histone alterations will affect response to therapy, particularly as numerous studies have shown an independent effect on clinical outcome and potential mechanisms of escape from therapy associated with these processes, and there is a need for retrospective analysis and prospective studies designed with these processes in mind.

## Data Availability

Not applicable.
